# Supplementary material for: Impact of a Withania somnifera and Bacopa monnieri Formulation on SH-SY5Y Human Neuroblastoma Cells Metabolism Through NMR Metabolomic
Source: Nutrients. 2024 Nov 28;16(23):4096. doi: 10.3390/nu16234096 (PMC11643393; doi:10.3390/nu16234096)
Supplement: Supplementary file 1 [file nutrients-16-04096-s001.zip › nutrients-3323006-supplementary.pdf]

Supplementary material

Impact of a *Withania somnifera* and *Bacopa monnieri* Formulation on SH-SY5Y Human Neuroblastoma Cells Metabolism Through NMR Metabolomic

Maria D'Elia [1,2,3], Carmen Marino [1], Rita Celano [1,2], Enza Napolitano [1], Anna Maria D'Ursi [1,2], Mariateresa Russo [4] and Luca Rastrelli [1,2,*]

[1] Department of Pharmacy, University of Salerno, Via Giovanni Paolo II, 132, 84084 Fisciano, Italy
[2] National Biodiversity Future Center—NBFC, 90133 Palermo, Italy
[3] Dipartimento di Scienze della Terra e del Mare, University of Palermo, 90123 Palermo, Italy
[4] Department of Agriculture Science, Food Chemistry, Safety and Sensoromic Laboratory (FoCuSS Lab), University of Reggio Calabria, Via dell'Università, 25, 89124 Reggio Calabria, Italy
* Correspondence: rastrelli@unisa.it; Tel.: +39-089969766

Table S1. Cross-validation performed with 10-fold methods reported the accuracy, R2 and Q2 value of three components.

Endometabolome	PC1	PC2	PC3
ACCURANCY	1.0	1.0	0.98
R2	0.89	0.87	0.76
Q2	0.91	0.88	0.67
Esometabolome	PC1	PC2	PC3
ACCURANCY	1.0	1.0	1.0
R2	0.92	0.94	0.80
Q2	0.78	0.85	0.65

Table S2. Metabolic pathways corresponding to serum and urinary metabolomic profile of intervention group. The pathways are classified according to p-values, Holm adjustment, and FDR values. The pathways are selected based on Hits > 2, Raw p < 0.05, and Holm adjust, FDR < 1.

	Hits	Raw p	Holm p	FDR
Fatty acid Metabolism	4	1.0617e-08	9.2364e-07	2.1662e-07
Mitochondrial Beta-Oxidation of Short Chain Saturated Fatty Acids	4	1.0617e-08	9.2364e-07	2.1662e-07
Mitochondrial Beta-Oxidation of Long Chain Saturated Fatty Acids	4	1.0617e-08	9.2364e-07	2.1662e-07
Glutamate Metabolism	10	1.2228e-08	1.0272e-06	2.1662e-07
Purine Metabolism	7	1,245E-05	1.0333e-06	2.1662e-07
Ketone Body Metabolism	2	2.9809e-08	2.4443e-06	4.3223e-07
Oxidation of Branched Chain Fatty Acids	4	4.3404e-08	3.5158e-06	4.7202e-07
Beta Oxidation of Very Long Chain Fatty Acids	4	4.3404e-08	3.5158e-06	4.7202e-07
Ammonia Recycling	9	8.0077e-08	6.3261e-06	7.7407e-07
Pantothenate and CoA Biosynthesis	5	1.1989e-07	9.3511e-06	0,001043
Glycine and Serine Metabolism	11	2.4921e-07	1.9189e-05	0,001971
Phosphatidylethanolamine Biosynthesis	3	6.0306e-07	4.5833e-05	4.2202e-06
Bile Acid Biosynthesis	4	6.6354e-07	4.9765e-05	4.2202e-06
Cysteine Metabolism	5	7.1355e-07	5.2803e-05	4.2202e-06
Amino Sugar Metabolism	6	7.4649e-07	5.4494e-05	4.2202e-06
Arginine and Proline Metabolism	8	7.7613e-07	5.5881e-05	4.2202e-06
Pyruvate Metabolism	7	1.1754e-06	8.3453e-05	4.7225e-06
Glycolysis	3	1.1942e-06	8.3593e-05	4.7225e-06
Galactose Metabolism	3	1.1942e-06	8.3593e-05	4.7225e-06
Lactose Synthesis	3	1.1942e-06	8.3593e-05	4.7225e-06
Lactose Degradation	3	1.1942e-06	8.3593e-05	4.7225e-06
Trehalose Degradation	3	1.1942e-06	8.3593e-05	4.7225e-06
Gluconeogenesis	4	1.3146e-06	8.5446e-05	4.9724e-06
Glutathione Metabolism	7	1.7021e-06	0.00010894	4.9946e-06
Steroid Biosynthesis	4	1.7797e-06	0.00011212	4.9946e-06

Nucleotide Sugars Metabolism	2	1.9519e-06	0.00012102	4.9946e-06
Inositol Metabolism	2	1.9519e-06	0.00012102	4.9946e-06
Sulfate/Sulfite Metabolism	2	1.9519e-06	0.00012102	4.9946e-06
Starch and Sucrose Metabolism	2	1.9519e-06	0.00012102	4.9946e-06
Fructose and Mannose Degradation	2	1.9519e-06	0.00012102	4.9946e-06
Mitochondrial Electron Transport Chain	2	1.9519e-06	0.00012102	4.9946e-06
Inositol Phosphate Metabolism	2	1.9519e-06	0.00012102	4.9946e-06
Phosphatidylinositol Phosphate Metabolism	2	1.9519e-06	0.00012102	4.9946e-06
Phosphatidylcholine Biosynthesis	2	1.9519e-06	0.00012102	4.9946e-06
Ethanol Degradation	4	2.0879e-06	0.00012102	5.1899e-06
Aspartate Metabolism	8	2.4217e-06	0.00012593	5.8524e-06
Urea Cycle	7	2.9959e-06	0.00015279	7.0444e-06
Sphingolipid Metabolism	4	3.7662e-06	0.00018831	8.6227e-06
Threonine and 2-Oxobutanoate Degradation	5	0,004503	0.00022065	9.5639e-06
Glycerolipid Metabolism	3	4.5071e-06	0.00022065	9.5639e-06
Phytanic Acid Peroxisomal Oxidation	3	4.5071e-06	0.00022065	9.5639e-06
Folate Metabolism	4	4.8865e-06	0.00022478	9.9798e-06
Beta-Alanine Metabolism	3	4.9908e-06	0.00022478	9.9798e-06
Tryptophan Metabolism	4	5.0473e-06	0.00022478	9.9798e-06
Pentose Phosphate Pathway	3	9.7083e-06	0.00041746	1.7971e-05
Riboflavin Metabolism	3	9.7083e-06	0.00041746	1.7971e-05
Thiamine Metabolism	3	9.7083e-06	0.00041746	1.7971e-05
Alanine Metabolism	5	1.1198e-05	0.00044793	2.0297e-05
Fatty Acid Biosynthesis	3	1.2421e-05	0.00048441	2.2053e-05
Selenoamino Acid Metabolism	5	1.4648e-05	0.00055662	2.5487e-05
Tyrosine Metabolism	3	1.5945e-05	0.00058995	2.72e-05
Malate-Aspartate Shuttle	2	1.9127e-05	0.00068857	3.2001e-05
Butyrate Metabolism	3	2.3165e-05	0.00080316	3.5356e-05
Mitochondrial Beta-Oxidation of Medium Chain Saturated Fatty Acids	3	2.3165e-05	0.00080316	3.5356e-05

Valine, Leucine and Isoleucine Degradation	8	2.4647e-05	0.00080316	3.6971e-05
Nicotinate and Nicotinamide Metabolism	5	0,02965	0.00080316	3.9678e-05
Phenylacetate Metabolism	4	3.0101e-05	0.00080316	3.9678e-05
Histidine Metabolism	4	3.0585e-05	0.00080316	3.9714e-05
Lysine Degradation	3	3.2962e-05	0.00080316	4.2172e-05
Warburg Effect	8	3.9826e-05	0.00080316	5.0215e-05
Propanoate Metabolism	7	4.2154e-05	0.00080316	5.2391e-05
Citric Acid Cycle	4	6.2666e-05	0.0010653	7.6788e-05
Methionine Metabolism	7	6.6535e-05	0.0010653	8.0396e-05
Transfer of Acetyl Groups into Mitochondria	5	6.8914e-05	0.0010653	8.2131e-05
Homocysteine Degradation	3	0.00015622	0.0018747	0.00017883
Phenylalanine and Tyrosine Metabolism	5	0.00021273	0.0022564	0.00023728
Pyrimidine Metabolism	4	0.001039	0.0093508	0.0011442
Glucose-Alanine Cycle	2	0.0015072	0.012058	0.0016391
Arachidonic Acid Metabolism	2	0.0030233	0.021163	0.0032472
